# Revolutionizing load harmony in edge computing networks with probabilistic cellular automata and Markov decision processes

**DOI:** 10.1038/s41598-025-88197-9

**Published:** 2025-01-29

**Authors:** Dinesh Sahu, Nidhi, Rajnish Chaturvedi, Shiv Prakash, Tiansheng Yang, Rajkumar Singh Rathore, Lu Wang, Sabeen Tahir, Sheikh Tahir Bakhsh

**Affiliations:** 1 SCSET, Bennett University, Plot Nos 8, 11, TechZone 2, Greater Noida, Uttar Pradesh 201310 India; 2 Department of Electronics and Communication, University of Allahabad, Prayag Raj, Uttar Pradesh India; 3 University of South Wales Pontypridd, Pontypridd, UK; 4 Cardiff School of Technologies, Cardiff Metropolitan University, Cardiff, UK; 5 Xi’an Jiaotong-Liverpool University Suzhou, Suzhou, China

**Keywords:** Edge computing, Resource scheduling, Load balancing, Probabilistic cellular automata, Markov decision processes, Load harmony, Computer science, Information technology

## Abstract

In general, edge computing networks are based on a distributed computing environment and hence, present some difficulties to obtain an appropriate load balancing, especially under dynamic workload and limited resources. The conventional approaches of Load balancing like Round-Robin and Threshold-based load balancing fails in scalability and flexibility issues when applied to highly variable edge environments. To solve the problem of how to achieve steady-state load balance and provide dynamic adaption to edge networks, this paper proposes a new framework that using PCA and MDP. Taking advantage of the stochasticity of PCA classification our model describes interactions between neighboring nodes in terms of a local load thus allowing for a distributed, self-organizing approach to load balancing. The MDP framework then determines each node’s decision-making with the focus on load offloading policies that are aligned with rewards that promote per node balance and penalties for offloading a larger load than it can handle.These models are then incorporated into our proposed PCA-MDP system to achieve dynamic load balancing with low variability in resource usage among nodes. By conducting a large number of experiments, we prove that the proposed PCA-MDP model yields a higher efficiency in the distribution of the load, higher stabilities of the reward function, and a faster convergence speed compared to the existing approaches. Key performance parameters, such as load variance, convergence time, and scalability, validate the robustness of the proposed model. Besides optimizing resource exploitation, load harmony in edge computing networks helps provide efficient work progression and minimize latency, thereby contributing to the advancement of the field with respect to real-time applications such as self-driving vehicles and the Internet of Things. The presented work offers an excellent foundation for the next-generation edge-computing load-balancing solution that can be easily scaled up.

## Introduction

Edge computing has emerged as a popular solution rapidly over the last few years and has already shifted the paradigm of data processing and storage. This change helps to reduce latency, improve performance and usability, and covers from IoT devices to real-time systems^1,2^. Nonetheless, enforcing efficient load balancing across these edge networks is difficult since the edge resources support a dynamic and distributed network infrastructure. It can be seen that conventional load-balancing strategies, including round-robin and threshold-based approaches, may not effectively meet sophisticated requirements for such tasks.

**Fig. 1:**
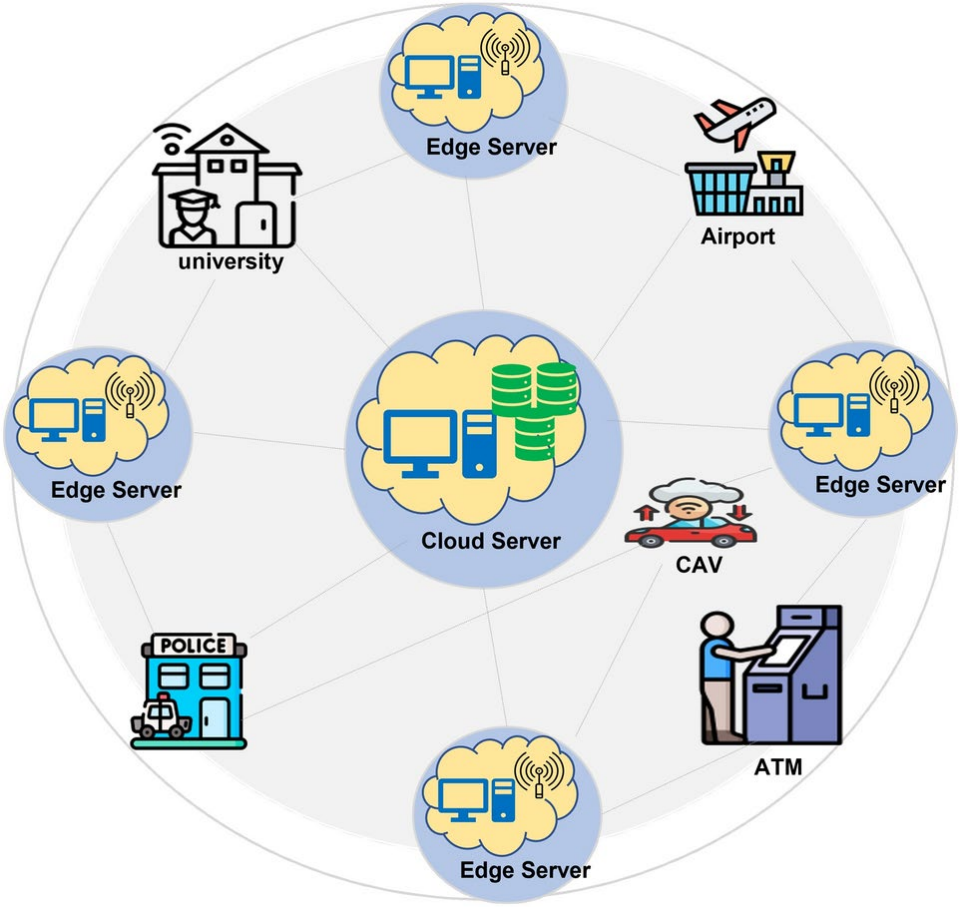
Edge computing.

Figure 1 depicts the general structure of the edge computing that highlights the position of the edge servers and cloud servers for various real-world applications. In the middle, there is used a cloud server that reflects intensive computational and storage on a single point, as it is type of distributed computing paradigm^1^. Around it are placed edge servers at proximity with data sources including universities, airports, police depots, and CAVs. These edge servers cut down the latency of operation by doing most of the computations locally, before pushing the data to the cloud; a feature strongly embraced in edge computing investigations^2^. Several use cases such as university systems, smart city applications including police and airport surveillance, and advanced vehicle operations (CAV) are shown to explain how Edge computing enhances real-time analytics, low bandwidth utilization, and efficient task execution^3^. This concept improves the system’s interactivity, robustness and expansiveness in several areas that were more or less inline with the industrial approach to handle issues related to latency-sensitive applications. Probabilistic Cellular Automata (PCA) and Markov Decision Processes (MDP) present some hopeful strategies for achieving adaptive load balancing in edge computing. As a distributed model based on cellular automata, PCA can handle probabilistic transition states in complex systems, with relatively high and low loads at the edge. On the other hand, MDP offers an environment in which the nodes are able to make decisions under the conditions of uncertainty be it in the context of the reinforcement learning under conditions of reward. This work therefore tries to overcome these shortcoming through the probabilistic model of PCA that can be achieved through MDP to balance load in real-time^4,5^.

### Motivation and challenges

Edge computing networks are leading crucial roles in supporting mission-critical and ultra-reliability demanded applications like the IoT, CAVs and AR systems. These networks make the computational resources deployment closer to the data sources hence enjoying low latency and excellent real-time response. Nevertheless, due to constantly changing workload, heterogeneity of resource capacities, and users’ unpredictable demand, these environments require effective mechanisms of load balancing to achieve high levels of system performance and reliability. For instance, the dissemination of edge computing across multiple end devices adds new challenges to the allocation of computational tasks while still ensuring minimal end-to-end delay and high system availability. These demands cannot be met by traditional centralized approaches because they can lead to scalability and latency problems that cannot handle the decentralized and resource-limited edge environments. Furthermore, since edge networks are extending to support more devices, the requirements of dynamic, sustainable, and simple load balancing solutions become critical. Besides, these solutions help for achieving the appropriate usage of edges resources, improve the user experience for minimizing the service disruption and ensuring the system can work stably under the various scenarios.

Edge computing networks experience some challenges that make it hard to balance the loads. Fluctuating and continuing loads arising out of real time application makes it nearly impossible to achieve load leveling. Moreover, resources of edge nodes are heterogeneous, may have different computational power, amount of RAM, or energy storage for completing tasks. Another problem is scalability since IoT and Edge devices are continuously proliferating, which requires load balancing services that can accommodate the growth and preserve low latency and high availability. In addition, real-time applications involve strict latency restraints where an answer must be delivered within a minor fraction of time that conventional load-balancing mechanics seldom achieve. Last, energy is important because for load balancing to occur, it has to balance it with energy and thereby increase the lifespan of the edge devices for sustainable use.

### Problem statement

Even though new load balancing algorithms have been developed to address some of the challenges in edge networks, most of the conventional load balancing approaches-such as Round-Robin, Threshold-Based, and Random Offloading-are not well suited for dynamic and distributed environments. They sometimes make assumptions about fixed resources, centralized management, or a known traffic load, which is worse when the network condition is dynamic. Traditional algorithms fail to handle the complexity of communication where in edge computing resources are scarce and fragmented into many nodes and nodes getting overloaded and resources being unutilized and tasks are taking a lot of time. This work aims at filling these gaps by developing load balancing model based on probability, PCA-MDP that will function under the network’s stochastic nature.

### Objective

The main focus of this paper is to propose and analyze load balancing model based on PCA and MDP for balancing the load in the edge networks between different nodes. The model will be able to adapt its distribution pattern based on probabilistic and reward based model to provide load balance to each node without having much focus on the central control system. By utilizing both PCA and MDP, the proposed model strives to outperform conventional load balancing techniques, particularly in complex and adaptive network scenarios.

### Contributions

The main contributions of this work are as follows:

1. *Development of the PCA-MDP model:* A novel load balancing model based on PCA and MDP that allows to take distributed decisions and adapt to diverse load scenarios in edge networks.In the proposed PCA-MDP model, probabilistic cellular automata offers the stochastic and localized load transition while MDP provides the decision-making and optimization. The integration also brings new novelties like the dynamic adaptation of the transition probabilities and a load balancing reward system that focuses on energy and latency rates.
2. *Comparative analysis:* A comparative analysis of the PCA-MDP model with traditional algorithms such as Round-Robin, random offloading, and the threshold-based model with respect to load variance, convergence, and resource utilization.
3. *Simulation and results:* A detailed discussion of the performance criteria highlighting how the model is effective in dealing with dynamic and distributed loads.

The rest of the paper is organized as follows: “Related work” section comprises the literature review which focuses on the load balancing in edge computing, defined as the load balancing process and the contemporary and conventional load balancing schemes. “Theoretical foundation” section is dedicated to the explanation of the proposed PCA-MDP model where its architecture and theoretical background are described. In “PCA and MDP load balancing model” section, the PCA-MDP load balancing model is introduced with theoretical background and design goals, followed by “Experimental setup” section which discusses the simulation setup, the parameters used in the experiment, and the evaluation metrics. Finally, based on the results, performance comparisons of the proposed PCA-MDP model with other methods is done in “Result and analysis” section. Lastly, “Conclusion and future scope” section provides the conclusion of the paper and possible direction for future research.

## Related work

Traditional load balancing has been extensively implemented in distributed computing platforms owing to its basic yet effective procedure for workload sharing. Round-Robin (RR), one of the first strategies, spreads tasks cyclically across the available nodes assigning each of them work in turn completely ignoring the status or capability of the node^6^. Although simple to use, RR has been found to cause suboptimal throughput especially under fluctuating workload because the scheduler does not consider when a node is heavily laden compared to others^7^. Random Offloading (RO) assigns tasks with high randomness which mean more flexible but no centralize decision making unit, that cause unbalanced load distribution in actual situation^8^. Threshold-Based Balancing (TB) allocates limitation of load capacity for each node and transfers tasks only if the load crosses these limitations^9^. This method provides some flexibility but generally is not suitable for complex and changing large scale systems where good thresholds are difficult to identify^10^.

Probabilistic Cellular Automata (PCA) have been used for network management for modeling decentralized systems based on probabilistic state transition that can make local decisions in wireless and distributed environment^11,12^. For instance, PCA has been utilized in the modeling of adaptive routing in wireless sensor networks and this is due to the observed flexibility in the design that can cope with node failure^13^. Likewise, Markov Decision Processes (MDP) is widely used for making decisions in a sequential manner under condition of risk, it has been applied in coordinating allocation of resources as well as scheduling of tasks within cloud infrastructure^14^. In one study, the authors used MDP based techniques to determine how to schedule different tasks with the efficient delay for multi-access edge computing systems^15^. However, the approaches discussed above commonly analyze PCA and MDP separately, failing to consider possible synergies between them with respect to adaptive, decentralized control^16,17^.

Despite the simplicity of the techniques such as RR, RO, and TB, they are insufficient to overcome the dynamics and heterogeneity of the considered edge computing setups^18,19^. Furthermore, it is found that the purely MDP or PCA-based strategies just emphasize on the part of load balancing, namely state transfer or reward optimization, and so it lacks comprehensive load balancing performance^20,21^. The strengths of using both probabilistic transitions and adaptive decision-making are described below. What it offers is real-time load balancing that depends on the current network conditions, thus increasing both the use of all resources available and the definite order of the tasks’ execution^22,23^. The PCA-MDP model also solves limitations on static configurations and slow convergence in compared techniques^24,25^, which makes the work a significant contribution towards improving load management in edge computing^7,26–34^.

### Maintaining the integrity of the specifications

The main focus of this paper is to propose and analyze load balancing model based on PCA and MDP for balancing the load in the edge networks between different nodes. The model will be able to adapt its distribution pattern based on probabilistic and reward based model to provide load balance to each node without having much focus on the central control system. The use of both PCA and MDP, as employed in the proposed model, will therefore serve as a tool of out-comping the current model of load balancing in complex and adaptive network environment.

## Theoretical foundation

For the development of PCA-based model for load balancing in edge computing, it is essential to understand certain basics of Probabilistic Cellular Automata (PCA). Incorporating PCA in the context of edge computing gives an approach to model systems where nodes (as edge devices) exhibit local and probabilistic interactions. These models can allow one to solve the load distribution problem by taking the probabilistic transitions between states with the help of the neighboring nodes, which interprets the dynamics of the task offloading through the network of the edge devices. The Table 1 contains the symbols and their descriptions used in this paper.

### Probabilistic cellular automata (PCA): basics and application to load balancing

**Table 1:** List of Symbols and Their Descriptions.

Symbol	Description
$S$	Set of states in the Markov Decision Process (MDP)
$A$	Set of actions available to nodes for load balancing
$P(s'|s, a)$	Transition probability from state $s$ to $s'$ given action $a$
$R(s, a)$	Reward function for taking action $a$ in state $s$
$\gamma$	Discount factor for future rewards in MDP (0 $\le$ $\gamma$ < 1)
$Q(s, a)$	Q-value representing the expected cumulative reward for state $s$ and action $a$
$\pi (s)$	Policy that determines the action to be taken in state $s$
$\alpha$	Learning rate in reinforcement learning algorithms
$T$	Total number of time steps or iterations
$N$	Total number of nodes in the edge computing network
$\lambda$	Task arrival rate in the edge network
$\mu _i$	Service rate of node $i$
$L(t)$	Load at a node at time step $t$
$p_{ij}$	Probability of state transition from cell $i$ to cell $j$ in PCA
$C$	Computational capacity of a node
$E$	Energy consumption in edge computing tasks
$f_i$	Offloading frequency for node $i$
$\sigma$	Standard deviation of load distribution among nodes
$\delta$	Convergence tolerance for the Q-value function
$\theta$	Threshold value used in threshold-based load balancing
$\eta$	Exploration-exploitation trade-off parameter in Q-learning
$\phi$	State transition matrix in PCA models
$\tau$	Time delay for task offloading
$\omega$	Weight assigned to specific criteria in decision-making
$M$	Maximum allowable load per node
$B$	Bandwidth available for data transmission
$\zeta _i$	Cost function for node $i$
$\rho$	Resource utilization efficiency
$k$	Number of states in a PCA neighborhood
$\beta$	Probability parameter for probabilistic state transition in PCA
$d_{ij}$	Distance between nodes $i$ and $j$ in the edge network
$\psi$	Penalty term for load imbalance
$\upsilon$	Utility function for resource allocation
$\chi$	Convergence metric for Q-value iterations
$\nu$	Control parameter for offloading decisions
$\mathcal {L}$	Loss function used in optimization algorithms
$\epsilon$	Exploration rate in exploration-exploitation trade-off
$\kappa$	Communication delay in edge computing
$\xi$	Factor for load adjustment across nodes
$\theta _{max}$	Maximum threshold limit for load balancing triggers

Cellular Automata (CA) are mathematical models to capture behavior of complex systems or objects with help of simple local rules implemented at each cell. In PCA, state transitions are probabilistic rather than forced allowing for adaptability for model systems such as edge computing environments that may vary dynamically. From a structural perspective, PCA is composed of a series of nodes that are placed on a grid to represent edge devices. In PCA, every node (cell) has

1. *A set of states:* States describe the load of each edge device as represented by the states of the different states. For example, each node can be described by one of several possible states in which it can exist at any given time: idle, moderately loaded, heavily loaded, or overloaded.
2. *Transition probabilities:* These describe how each state can change with time given the current state of the node and the state of other connected nodes.
3. *Neighborhood interactions:* Nodes change their state according to the state of their neighbors based on the defined neighborhood radius which represents how loads in nearby devices affect the neighbors devices. In edge computing, every node only deals with a few neighboring nodes, making neighborhoods that determine load distribution.

**Fig. 2:**
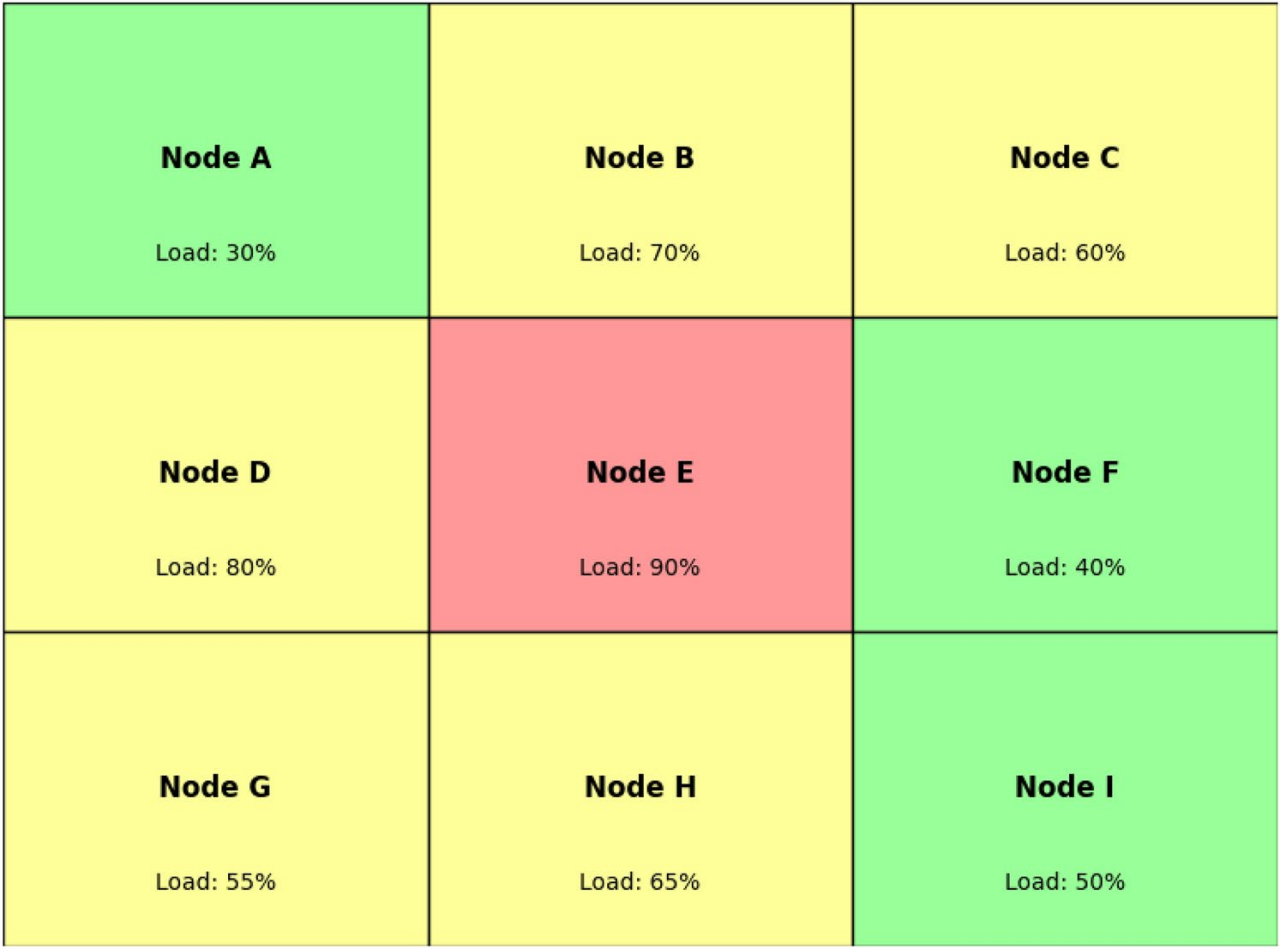
Overview of PCA model for load balancing in edge computing.

In Fig. 2, there is a 2D grid where each node is connected to the nodes in their neighborhood. The load percentages reflects the current load status of each node, which varies randomly according to its load status of neighboring nodes. For instance, if Node E is overloaded the latter may try to redistribute them to less congested neighbors, namely: Nodes B, D, F and H in a probabilistic fashion.

In PCA Each node $i$ at time $t$ is in a specific load state $s_i(t)$, where: $s_i(t) \in \{0, 1, 2, \dots , S\}$. $S$ represents the maximum load state. For simplicity, we may define $0$ as idle, intermediate values as partially loaded, and $S$ as overloaded. The probability at which a node may change from one state to another is influence by its current state and that of its neighborhood nodes. Let $P_{i,j}$ represent the probability of node $i$ transitioning to state $j$:

1 $$\begin{aligned} P(s_i(t+1) = j \mid s_i(t), N_i(t)) = f(s_i(t), N_i(t)) \end{aligned}$$

where $N_i(t)$ represents the neighborhood states around node $i$ at time $t$.As for the transition probability function $f$, it will allow having more flexibility in order to obtain the desired load balancing logic. For instance, a node in a high load level, such as 3, is likely to transit to a low load level if other neighboring nodes are at low class load levels.

**Fig. 3:**
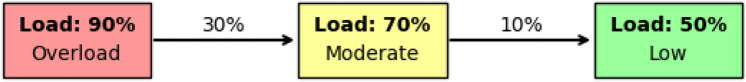
Transition probability.

Figure 3 shows a simple representation of transition probability in load balancing. In case of high load node (for instance 90%), there is a probability (30%) to offload jobs to a node with moderate load and a small probability (10%) for low load node.Each node computes the transition probability based on the state of neighbors to recognize their transition from one state to another. If a node is overloaded, the probability of it offloading tasks to a neighbor depends on the load state of that neighbor:

2 $$\begin{aligned} P(s_i(t+1) = j) = \frac{1}{|N_i(t)|} \sum _{k \in N_i(t)} \left( 1 - \text {Load}(s_k(t))\right) \end{aligned}$$

This function takes into account the load of each neighbor with neighbors that have lower load having a higher impact on the probability of offloading a task.The PCA model progresses over the successive time steps where the node state is probabilistically updated based on local neighborhood. At some point in the future, the load distribution becomes stochastic and none of the nodes continues to get overloaded beyond that point indicating fairly load distributed in the network.

**Fig. 4:**
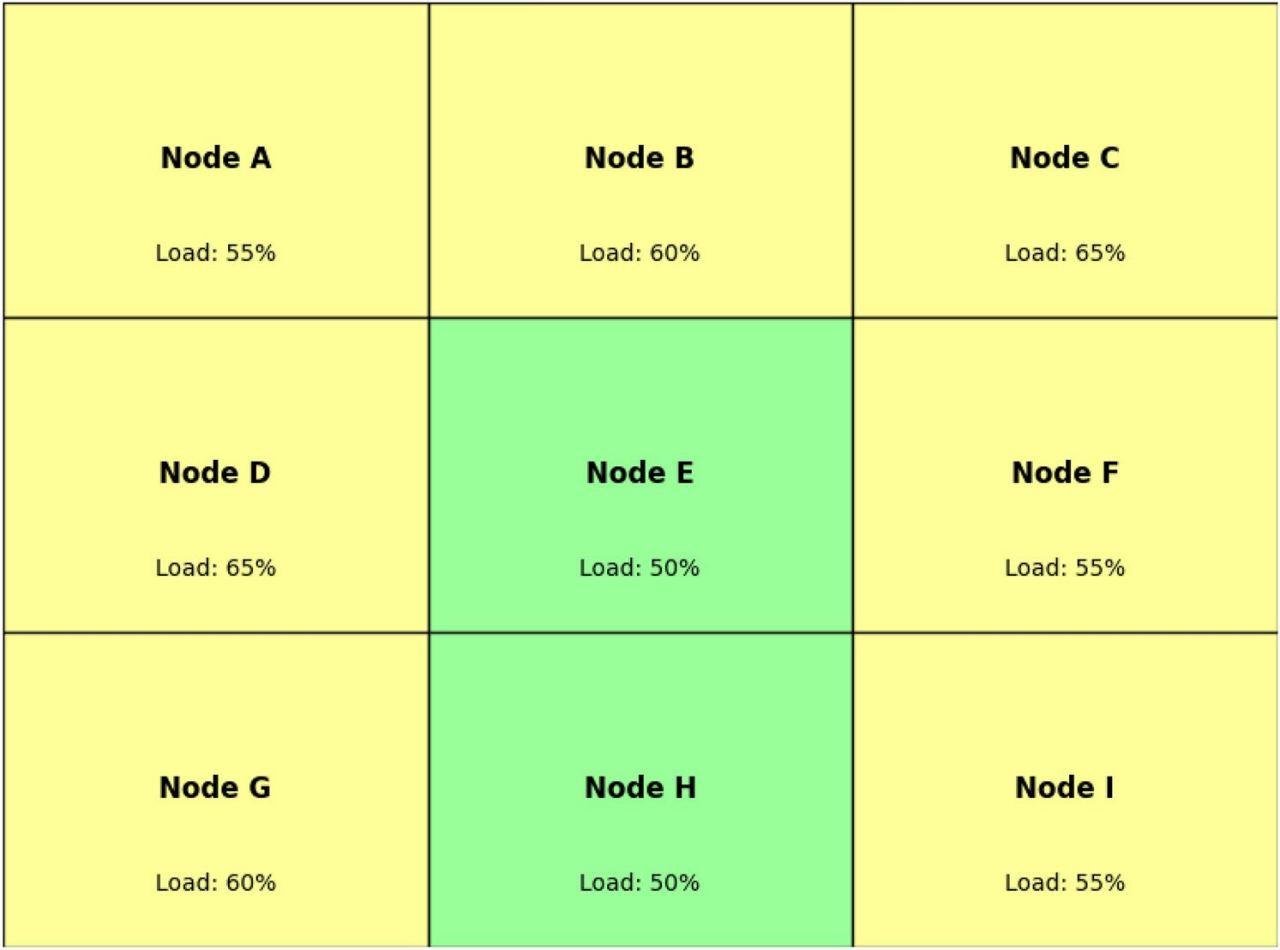
System equilibrium achieved through PCA-based load balancing.

As shown in Fig. 4, after several iterations of the PCA model, load is distributed evenly among nodes; no node becomes a bottleneck or overloaded It can be seen that the PCA is a highly suitable model for enabling adaptive, distributed load balancing in edge computing, with the ability to adapt in real-time to changes in node status, as well as interactions and dynamics among nodes. Because PCA is probabilistic, edge devices can distribute loads well while avoiding centralized control, making it possible to develop a robust load-balance solution for complex distributed edge networks.

### Markov decision process (MDP)

Markov Decision Processes (MDPs) is a formal model that can be used to solve decision-making problems whenever the decision maker faces probabilistic outcome, that is a mix of randomness and decided outcome. MDPs are most useful in load balancing in edge computing where the intent is to distribute workload across edge devices taking into account uncertainties of load patterns and resource availability.

In MDP the state $S$ define the possible configurations of the system in the given moment of time. In the case of edge computing, a state can mean the load status of every edge device that is connected to the network. For example: $S = \{ s_1, s_2, s_3, \ldots , s_N \}$ where each $s_i$ is a specific load state (e.g., idle, moderate load, high load) of an edge device.

Action is simply a decision that the decision maker has at their disposal when they are in any given state. For load balancing, available actions can be to hand over to neighbor devices, to redistribute work with neighbor device or to simply reject a load change request. The action space can be represented as $A = \{ a_1, a_2, a_3, \ldots , a_M \}$.

In the context of reinforcement learning, rewards put a numerical value to the immediate payoff that one derives by performing a given action in a specified state. In edge computing, the reward can be defined based on factors such as task completion time, resource utilization efficiency, or energy consumption: $R(s, a) \rightarrow \text {Reward received after taking action } a \text { in state } s$ A transition probability actually gives the probability of moving from one state to another when an action is taken. They define the dynamics of the system:

3 $$\begin{aligned} P(s' \mid s, a) = P(s_{t} = s' \mid s_{t-1} = s, a) \end{aligned}$$

This notation means the probability of moving to state $s'$ from state $s$ after taking action $a$.

**Fig. 5:**
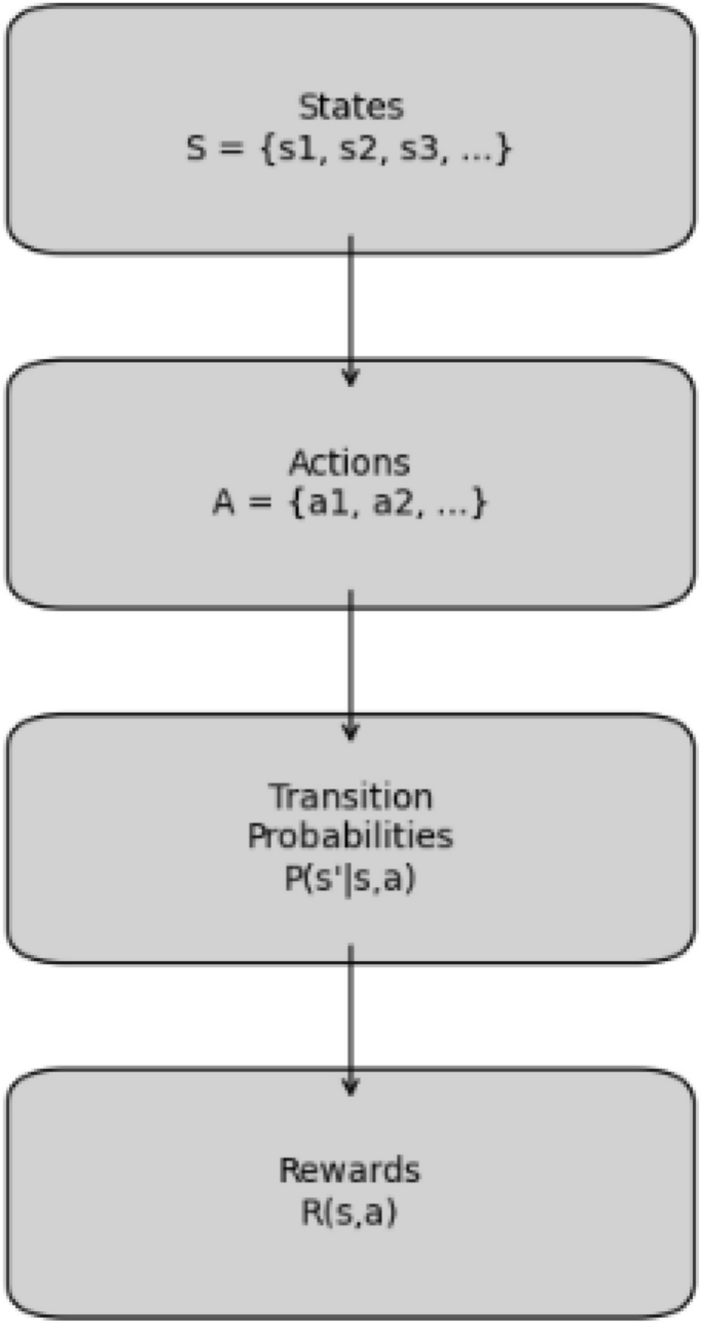
Overview of MDP components.

Markov Decision Processes (MDPs) provide an improvement in decision-based load balancing by way of these key processes as represented in Fig. 5. First, a policy specifies how an action is chosen for each state, written as $\pi : S \rightarrow A$ This policy works in context of load balancing, where it defines a geometrical distribution of a given task based on the load at edge devices. Another important ingredient is the Q function assessing the expected total lifelong reward of each state in the course of running of the policy.

4 $$\begin{aligned} V(s) = \mathbb {E} \left[ \sum _{t=0}^{\infty } \gamma ^t R(s_t, a_t) \right] \end{aligned}$$

where $\gamma$ is the discount factor which defines how future rewards matter.s. A higher value shows that the long-term load distribution is more beneficial. Finally The goal is to find the optimal policy $\pi ^*$ that maximizes the expected rewards:

5 $$\begin{aligned} \pi ^* = \arg \max _{\pi } V(s) \end{aligned}$$

In the context of edge computing, the optimal policy plays a crucial in defining how the tasks should be partitioned across edge devices with respect to load balancing.

**Fig. 6:**
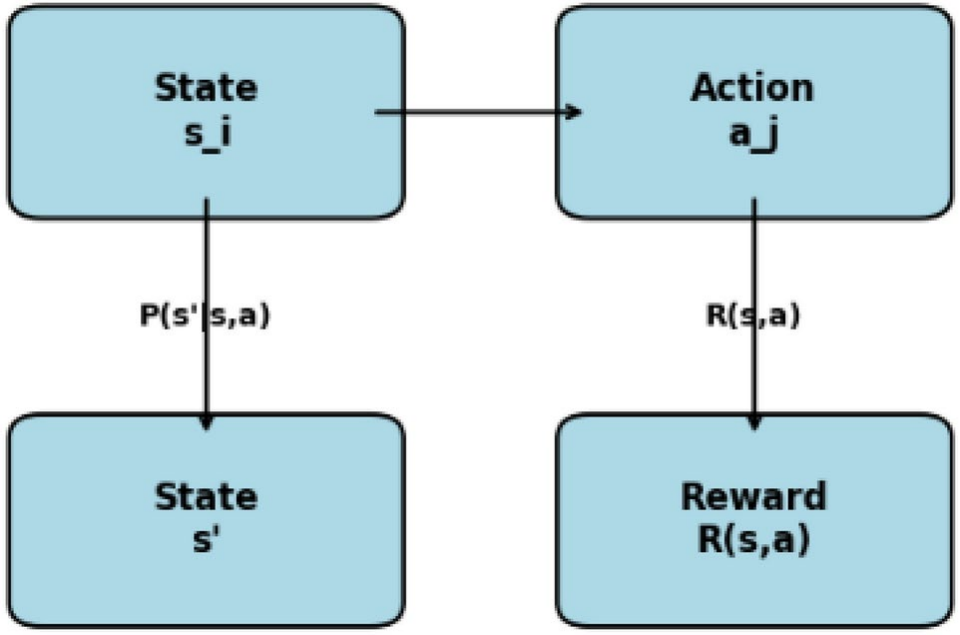
Decision-making process in MDP for load balancing.

Moreover, in the context of load balancing applications, it is shown that MDPs can be used in edge computing through task offloading and resource allocation in a readily dynamic manner. In terms of task offloading, each edge device considers its own and its neighbors’ current loads. When in a high load status, one may opt for offloading tasks to relatively idle neighbors. This decision making process is amenable to MDP modeling; the offloading transition, redistribution transition or the state stay decision transitions - all of them affect the state evolution and the reward prospects. Similarly, in case of dynamic resource allocation, since tasks are processed on a continuous basis, edge devices must frequently re-evaluate their actions as per them and their neighboring devices’ state. Through MDPs, the devices perform what action based on the states that would yield the best long-term results such as decreased overall latency times or even power consumption, The same has been depicted in Fig. 6.

**Fig. 7:**
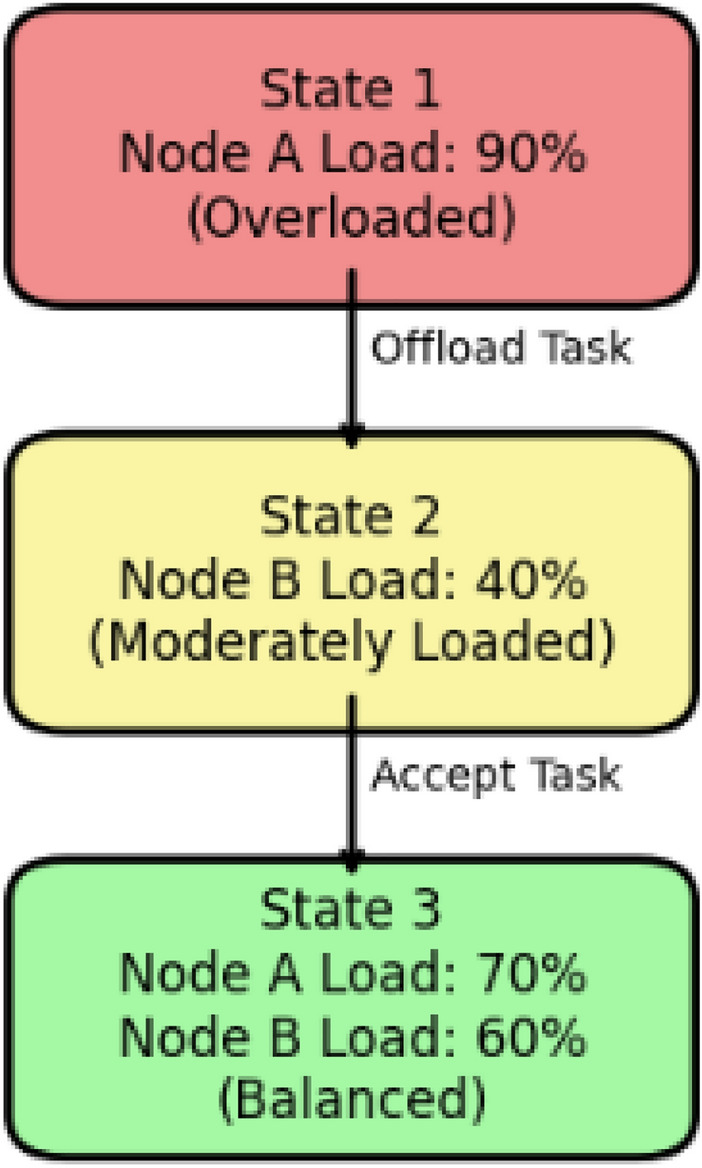
MDP in action for edge computing load balancing.

In Fig. 7, Node A is experiencing a high load and opts to transfer workloads to Node B that is experiencing a moderate load. The step taken results in a new condition which improves the load distribution somewhat among the two nodes.

Markov Decision Processes provide a formal approach to load balancing decision making in the context of edge computing. Through the specification of states, actions, rewards and transition probabilities, MDPs enable edge devices to act in a way that improves performance and efficiency. By employing MDPs, adaptive resource allocation can be achieved and dynamic workloads across distributed systems can be managed effectively. By applying MDPs to the load balancing algorithms in edge computing, not only is operationally more efficient, but the entire system is more resilient and responsive.

The dynamic workloads are captured by the Probabilistic Cellular Automata (PCA) framework that defines the ability of edge nodes to move between load states based on their neighbors’ state with some probability. This end-to-end local interaction mechanism guarantees workload adaptation on a peer-to-peer basis as performed within a system, with no need for a controller. For instance, an overloaded node may probabilistically shift responsibilities to other less congested nodes, in order to achieve a dynamic load balancing in real time fashion. This characteristic of PCA makes it rather applicable to the edge networks with workload fluctuations of unpredicted characteristics.

The system used by this MDP includes a reward function that considers the properties of nodes, including computational power, memory resources, and energy. The integration of the above factors enable load balancing decisions to be bounded by the capabilities of these MDP edge nodes. Although such heterogeneity brings in system flexibility this design ensures that a certain resource is not overused and at the same time optimizes the overall efficiency of the whole edge network.

Due to the decentralized nature of PCA, the model can be easily extended for larger networks if needed. Here each node communicates only with its bounds neighborhood neighbors resulting in less complexity and hence the system scalability even with large edge networks. Moreover, the reinforcement learning process in MDP adapts policies based on the structures in the network which makes the implementation suitable for large networks.

In general, the hybrid PCA-MDP model aims at reducing latency through achieving the goal of task redistribution through PCA and efficiently determining task offloading policies through MDP. Moreover, reward design in MDP provides further encouragement for energy-saving choices while achieving low latency. Such a dual focus of the model guarantees that the model is effective in handling latency-sensitive and energy-consuming edge computing applications.

In general, the hybrid PCA-MDP model aims at reducing latency through achieving the goal of task redistribution through PCA and efficiently determining task offloading policies through MDP. Moreover, reward design in MDP provides further encouragement for energy-saving choices while achieving low latency. Such a dual focus of the model guarantees that the model is effective in handling latency-sensitive and energy-consuming edge computing applications.

## PCA and MDP load balancing model

Integrating Probabilistic Cellular Automata (PCA) and Markov Decision Processes (MDP) offers a highly efficient model for probabilistic load balancing in edge networks. This integration exploits the local probabilistic interactions with the PCA and the global rigorous process of making decisions with the help of MDP. Hybridizing between traditional models of reinforcement learning, edge nodes can adapt the offline and online offloading strategy as well as resource assignment dynamically according to the load states of the edge nodes and their neighbors, as well as learning the best actions in their lifelong learning process.The PCA-MDP model presented in this work integrates local probabilistic relations of PCA with the structured and decision based optimization of MDP for optimal load balancing in edge computing networks. This integrated model can allow each edge node decide whether to offload its tasks or not depend on load state, the network structure as well as historical reward feedback which leads to load balancing.

### System architecture

The load balancer in the newly proposed PCA-MDP model is a preliminary novel hybrid scheme for the load balance in edge computing environment that integrates the localized learning of the Probabilistic Cellular Automata (PCA) as well as the global optimization characteristics of the Markov Decision Process (MDP). Unlike other approaches that only use fixed models with a deterministic structure or reinforcement learning structures like the Deep Q-Network (DQN), PCA-MDP employs PCA’s stochastic transition mechanisms to redistribute workloads in neighboring nodes. This allows for real time control in response to variations in load and heterogeneity of resources in a workforce. The MDP component on the other hand uses a task-specific reward function and integrates load variance, energy consumption and latency requirements towards its reward structure. This integration ensures that local task redistribution fulfills the general goal of the global system to provide optimal load balancing for efficient functioning in dynamic edge network circumstances.As for the proposed PCA-MDP compared to some of the SOTA methods like GA and PSO, it shows a number of advantages. In most cases SOTA methods have been found to use heuristic or optimization methods whereas PCA-MDP strikes a good balance between centralized and decentralized decision making. This hybrid design enables it to grow in a large edge network and offer high resource use and rapid convergence.The PCA-MDP Model of Edge computing network in has the following Architectural modules:

1. *Edge nodes:* The devices that can perform computations either locally or transfer these calculations to other nodes. Every node delivers information about its present load, available capacity, and load status of the adjoining nodes.
2. *Tasks:* These are on-the-fly required processing tasks like IoT data, frames for analysis, and real-time sensor data. All these tasks come with specific needs they possess for instance computation power and the bandwidth.
3. *Network structure:*The Network Structure is represented by a graph; the vertices are edges; the edges, in this case, illustrate link connections. These connections allow localized interaction and help to decide the offloading of tasks.

**Fig. 8:**
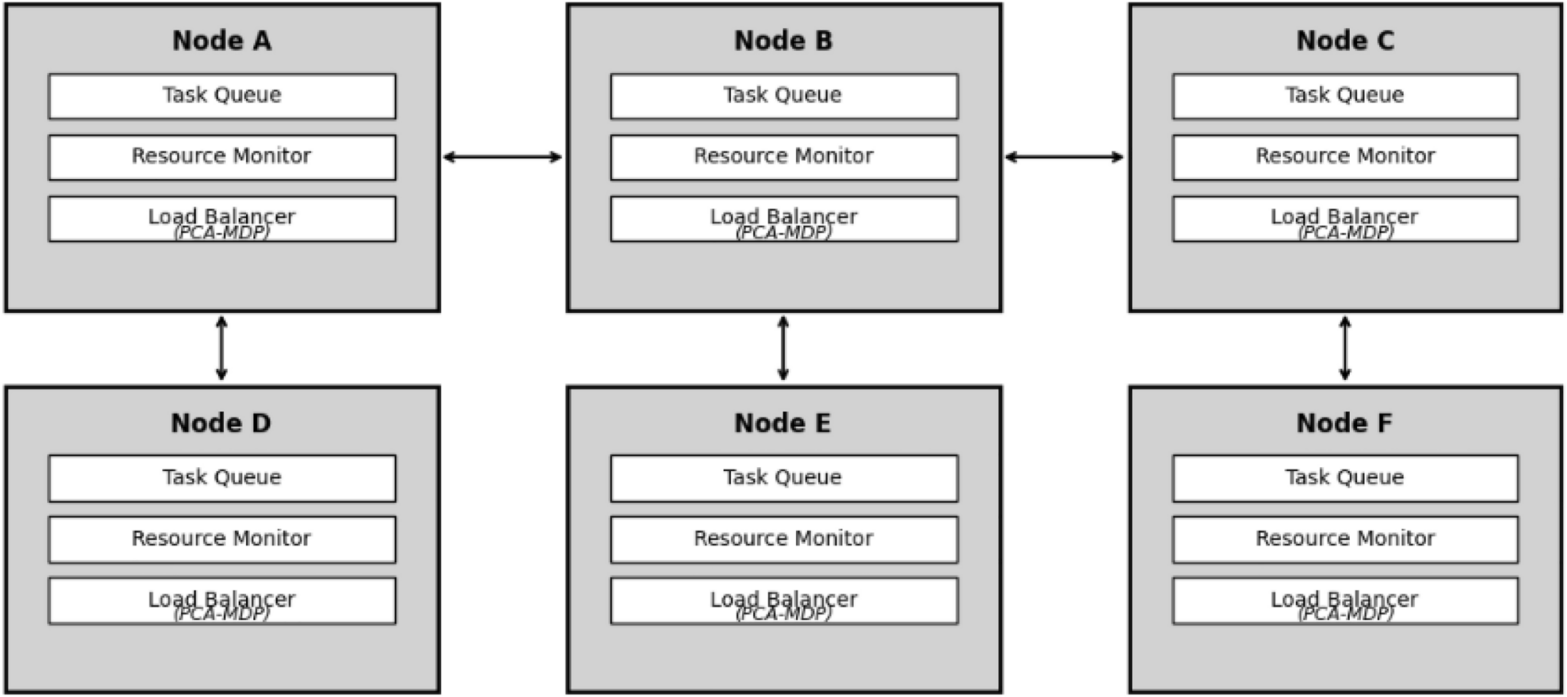
Edge network system architecture.

The Fig. 8 shows the structure of an edge computing network with PCA-MDP for load balancing. The architecture of each edge node (A, B, C, D, E, F) contains Task Queue, Resource Monitor, and Load Balancer with sub-modules for PCA and MDP of dynamic offloading. The nodes interact with their neighbors to exchange load states and to coordinate the load shifting according to the prevailing conditions. This architecture allows for dynamic load balancing due to the adaptation in the workloads and management of the network resources.Each node possesses information of its own status (loads, number of tasks, etc.) as well as status of the neighboring nodes. From this information, the different nodes are able to make adaptive load balancing decisions.

### PCA-MDP integration

Important aspects of PCA MDP integration are:

1. *State representation:* In this combined model, each node in the network corresponds to a cell in the PCA where the state of the node or edge device is determined by the current load of the cell such as idle cell, moderately loaded and highly loaded. The network state is defined as the array of node state which conveys the load snapshot of the whole edge network. All types of state transition that occur in the context of the PCA, including the load-level change due to the task’s completion, offloading, or resource reallocation, are used in the context of an MDP input state.
2. *Action decision making:*In the MDP framework, each node i decides its next action, according to dynamic load state of node i and other neighboring nodes. Activities performed in this PCA-MDP model may include transferring the tasks to a particular neighboring node, holding the task if the workload at the local node is low, or moving tasks among neighboring nodes to equalize load. In PCA transition probabilistic is used which determines the likelihood of a node to switch into lesser load state from its neighboring nodes and the MDP has rewards for the action which helps in load balancing.
3. *Reward structure:*The reward structure in MDP assists edge nodes to understand which of the performed actions contribute to achieving the best load balance in the future and reduce latency, energy consumption or time for a particular task. Outcomes of load balancing actions are associated with rewards since reward programs are tied to performance. For instance, an action giving an even distribution of load to the nodes receives high reward whilst actions that contribute to overloaded neighbors are punished.
  MDP uses the probabilistic transition of PCA to keep track of workloads of various edge nodes and adjust these probabilities to ensure that none of the nodes are overloaded while serving dynamic workloads. While PCA guarantees adaptive movement among load states, the MDP framework assesses the movement among them concerning their effects on the system performance creating a unique layered approach of managing dynamic workloads. In addition, the proposed PCA-MDP integration takes the heterogeneity of edge nodes into account through combining the local offloading ability of PCA with the reward-based mechanism of MDP to make the best of the energy, computation power and latency for different nodes. The efficiency is another essential advantage of PCA as a decentralized algorithm that does not exacerbate the computational demands of large networks and MDP that is capable of acquiring global policies over time adjusting to the growth of the network without considerable overhead. Last but not least, the hybrid model has dealt with latency and energy efficiency problems by incorporating PCA’s fast distribution of tasks within local neighborhoods and integrating MDP’s energy-efficient decisions. This dual condition also helps to minimize latency and energy utilization, which implies the model could be appropriate for real-time application in IoT, AR, and CAVs.

### PCA MDP algorithm

**Fig. 9:**
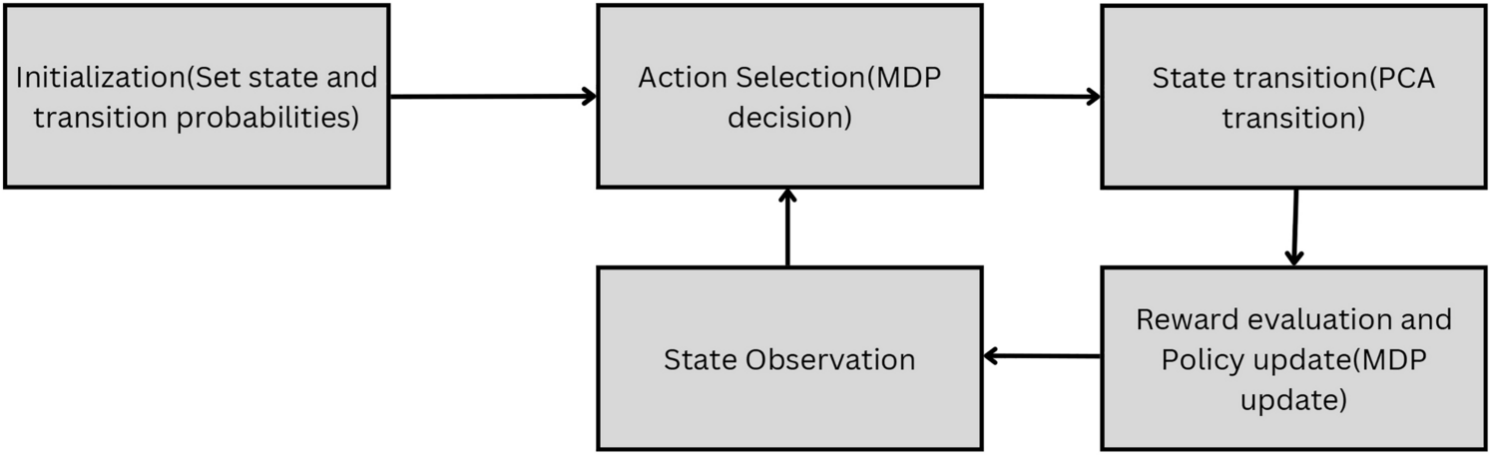
PCA-MDP algorithm process flow.

The PCA-MDP algorithm works in a step-by-step manner and consequent to adoption of local control and learning control for equal distribution of load across the network. Each iteration includes four key stages as per Fig. 9.

1. *Initialization:* Every node begins the sample data at some load state (idle, moderate, highly loaded). It also follows states of its neighbor nodes. In the PCA model, the probabilities in the state transition processes are determined by the loads of the neighboring nodes. For example, if most nodes have high loads, a node loading has high probability of offloading tasks.
2. *Action selection:* Every node assesses its load state and perform a particular action based on the MDP policy where states correspond to the actions. Possible actions may include transferring tasks to a neighbor with a lower load, dividing the load with several neighbors or keeping the tasks if the local load is low. The selected action is the one that is anticipated to give the most significant reward in regard to the previous rewards, along with the load condition of the adjacent nodes.
3. *State transition:* When a node has chosen an action, its load state changes and propagates to the other nodes in the network in accordance with the precautions of PCA transition. Transition probabilities reflect the probability of transitioning from the current state to a new state based on the action taken.Probabilities are influenced by the state of neighbor nodes which is an additional level of flexibility, when considering load balancing.
4. *Reward function and policy update:* Subsequent to the state change, every node assesses a reward according to load-balance requirements (for example, the resource usage or the time taken to complete tasks).Rewards R(s, a) inform action effectiveness to the nodes and enable the update of the MDP policy. This to ensure the best balance of load at any given time in the future is achieved since the policy update process is meant to give the highest future expected reward.
5. *State observation:* Following reward evaluation and resulting policy changes, the node becomes aware of its state encompassing current load and the status of other nodes. This observed state is then fed into the next part of the action selection to check whether the current action selected is appropriate given the nodes current state and the updated policy.

### Mathematical model

Developing a mathematical model of a balanced load distribution based on PCA and MDP for edge computing implies the identification of basic concepts of the mentioned models, definition of the transition functions and states; rewards and learning mechanism. In the following sections, lay out of this model is given in detail.

Each edge node i in the system can be considered as a “cell” of the PCA model, which is in contact with its nearest neighbors.Let $N_i$ be the set of neighboring nodes of node $i$. Each node has a current load $L_i$, which can be formulated as:

6 $$\begin{aligned} L_i = \frac{\text {Current Load of Node } i}{\text {Capacity of Node } i} \end{aligned}$$

The load $L_i$ can be categorized as Under-loaded (UL) if $0 \le L_i < 0.3$, Balanced (BL) if $0.3 \le L_i < 0.7$ and Overloaded (OL) if $0.7 \le L_i \le 1.0$. Specifically, thresholds at 0.3 and 0.7 are chosen from related practices in resource utilization research and have been empirically tested through simulation protocols. These thresholds define underloaded, optimum and overloaded states which help in balancing loads and avoiding unjustifiably low Performance. In the model, these thresholds can also be adjusted according to the needs of a particular application and that is why it is a flexible model.The objective of the load balancing model is to get each node in the network to a state of BL without the necessity of the high use of the state information. Probabilistic rules are used in PCA approach so that each node can independently make load-balancing decision based on its state as well as the states of its neighbors. These are called the transition probabilities and they represents functions that determine the probability of load offloading/acceptance actions.In the case of two adjacent nodes i and j the probability $P_{ij}$ denotes the chance that node i will transfer some of its burden to node j. We can define this probability based on the load difference between the nodes:

7 $$\begin{aligned} P_{ij} = \frac{1}{1 + e^{-\beta (L_i - L_j)}} \end{aligned}$$

where $\beta$ is a tunable parameter controlling sensitivity. Higher values of $\beta$ make the transition probability more sensitive to load differences. $L_i$ and $L_j$ are the load states of nodes $i$ and $j$.This sigmoid function ensures that When $L_i > L_j$, $P_{ij}$ is high, encouraging load offloading from node $i$ to node $j$ and when $L_i \le L_j$, $P_{ij}$ is low, discouraging unnecessary load shifting.

Each node $i$ transitions probabilistically based on the states of its neighbors $N_i$: if node $i$ is in an Overloaded (OL) state then it chooses to probabilistically perform load offloading to its neighbors with Under-loaded (UL) or Balanced (BL) state. When node $i$ is in an Under-loaded (UL) state it can also load from neighbors that are in an Overloaded (OL) state. If node $i$ is in Balanced (BL) state, it normally keeps its load, but can probabilistically lighten or receive tiny load to achieve load balance of relative nodes.

To enhance these probabilistic transitions, we propose an MDP model that would allow each node to learn from earlier decisions while seeking to achieve a reward that corresponds to efficient load distribution.The state $s_i$ of each node $i$ is defined by its load $L_i$ and the load states of its neighbors $N_i$. For example,

$$\begin{aligned} s_i = (L_i, L_{N_1}, L_{N_2}, \ldots , L_{N_k}), \end{aligned}$$

where $L_{N_j}$ represents the load of each neighboring node $N_j$. The three choices available to each node are - offload, retain and accept load. “Offload” means whether the node $i$ is willing to offload some of the tasks to an adjacent node, “retain” signifies that node $i$ does not want any change occurring to its load, and “accept load” means node $i$ is willing to accept load from an adjacent node. Therefore, it can be understood that in the present MDP context of the traffic network, the transition probabilities change according to the actions performed by each node. For example,

8 $$\begin{aligned} P(s' \mid s, \text {Offload}) = P_{ij} \end{aligned}$$

where $P_{ij}$ represents the shifting the load from node i to node j based on the is the probability difference in load and states of adjacent nodes.Rewards are applied to address states that created balanced loads. For instance, a high reward is given when a node gets or remains in the balanced load state, while a penalty is given when a node enters or causes others to enter the overloaded state due to offloading.

9 $$\begin{aligned} R(s, a) = {\left\{ \begin{array}{ll} +1 & \text {if } 0.3 \le L_i < 0.7 \\ -1 & \text {if } L_i \ge 0.7 \text { or } L_j \ge 0.7 \end{array}\right. } \end{aligned}$$

Every node can acquire an optimal policy for the optimal amount of reward using a Q-learning algorithm. Define the Q-value function Q(s, a) for each state-action pair:

10 $$\begin{aligned} Q(s, a) + = \alpha \left( R(s, a) + \gamma \max _{a'} Q(s', a') - Q(s, a) \right) \end{aligned}$$

where $\alpha$ is the learning rate, $\gamma$ is the discount factor for future rewards and $\max _{a'} Q(s', a')$ represents the maximum expected future reward from the next state $s'$. Nodes update the Q-values and correct their policies of load balancing, to optimize the cumulative reward of their actions on a more ongoing basis.Regarding policy con vergence, we assume that each node converges to policies that optimize af reward function among various states based on the probabilistic transition. This occurs when:

11 $$\begin{aligned} \lim _{t \rightarrow \infty } Q(s, a) = Q^*(s, a) \quad \forall s, a \end{aligned}$$

where $Q^*(s, a)$ is the optimal Q-values for directing the policy for load balancing.

### Problem formulation

An optimization problem is framed for the load balancing problem in edge computing networks with objective function consisting of minimizing load variance ($\sigma _L$) while considering constraint of energy consumed ($E$) and time to complete tasks ($T$). The objective function can be expressed as:

$$\begin{aligned} \text {Minimize } F = \alpha \sigma _L + \beta E + \gamma T, \end{aligned}$$

where $\alpha$,$\beta$, and $\gamma$ show the proportionate weights of these objectives. This optimization is subject to the following constraints:

Node Capacity Constraint: $L_i \le C_i$, where $L_i$ is the load on node $i$ and $C_i$ is its capacity.

Latency Constraint: $T_{ij} \le \tau$, ensuring that task offloading between nodes $i$ and $j$ meets latency requirements.

Energy Constraint: $E \le E_{\text {max}}$, limiting total energy consumption. 

**Algorithm 1:**
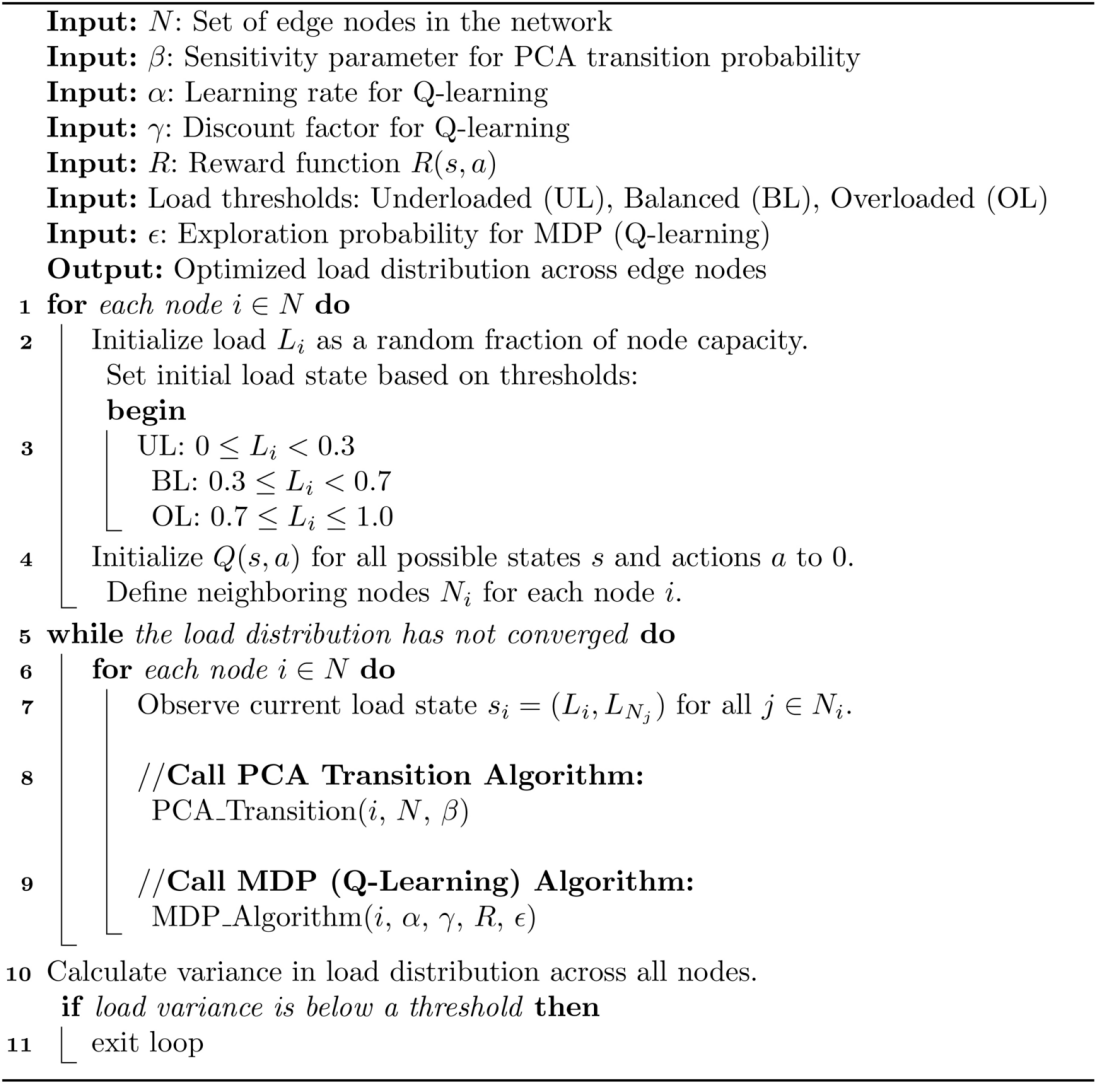
PCA-MDP load balancing for edge computing.

**Algorithm 2:**
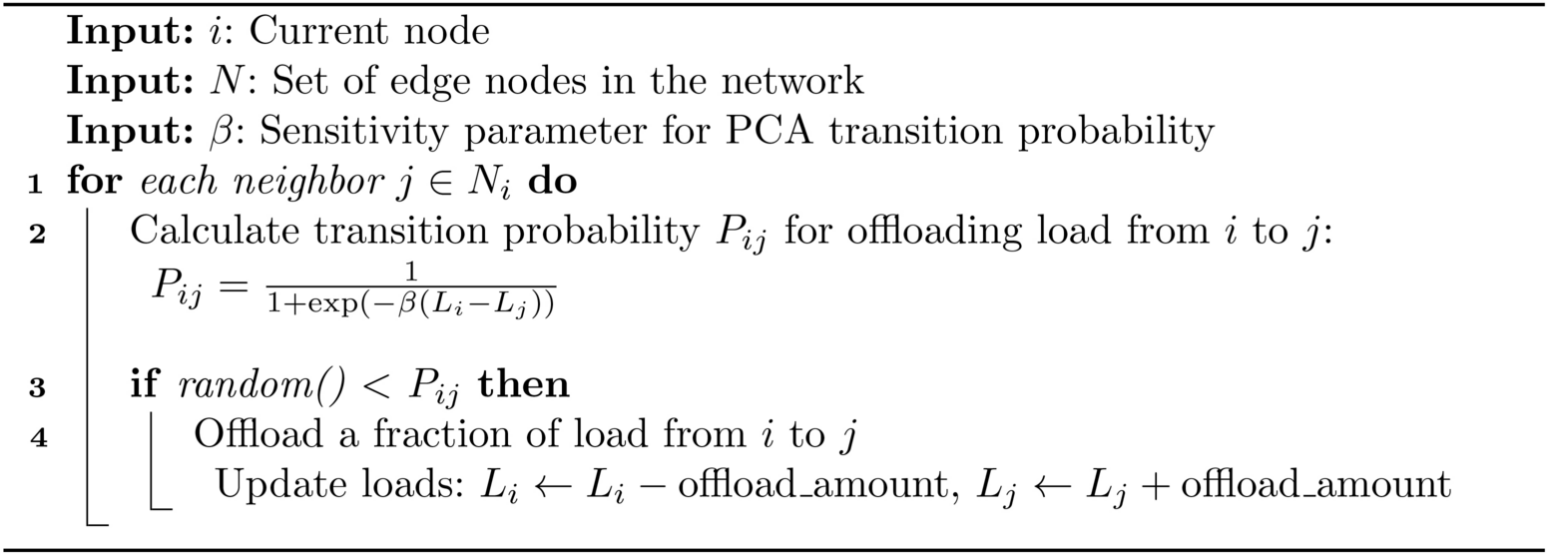
PCA transition for load balancing.

**Algorithm 3:**
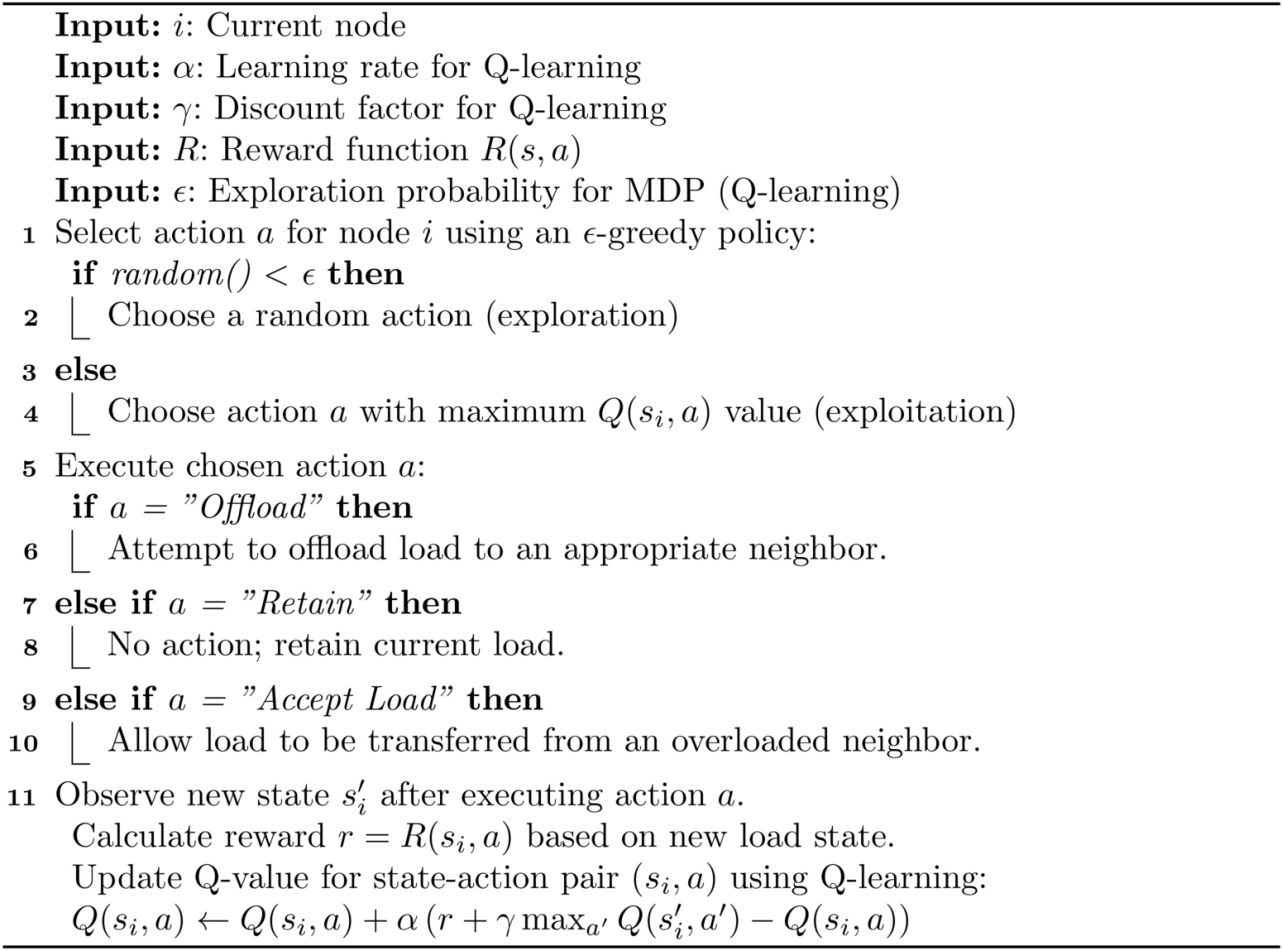
MDP for load balancing.

## Experimental setup

To test the effectiveness of the PCA-MDP model for load balancing in edge computing a controlled experimental setup, state-of-the-art benchmark algorithms, and a set of evaluation metrics are utilized. The proposed PCA-MDP model is evaluated in a real-like edge computing scenario where the network topology is reproduced realistically. Algorithm 1 is the main hybrid PCA-MDP algorithm which also uses Algorithm 2 (for PCA transition probability calculation) and Algorithm 3 for MDP calculations. Key characteristics of the environment include:

1. *Network size:*A simulation of a small to large scale infrastructure is created through the use of a network with 50-100 edge nodes. Every each node is directly connected to a several neighboring nodes in the same mesh structure that can be beneficial for the localized communication and for offloading of tasks.
2. *Task characteristics:*The tasks are created with different levels of computational complexity which creates variations across CPU usage, memory demands, and bandwidth. We consider the arrival of tasks as Poisson processes to capture variability and randomness in the number and intensity of tasks, which is typical for IoT and Edge computing. The tasks’ priorities and time frames are also different to understand how the load balance model functions depending on the degree of urgency.
3. *Computational resources:*Every node of the network is routined in term of computational power and storage, keeping in view the mode of commonly used edge devices like gateways or miniature servers. They are configured with CPU frequency in the range 0.5 GHz-2 GHz and RAM of 512 MB-8 GB, which gives a realistic model of the edge nodes with low and high capabilities.
4. *Communication cost:*Latency and bandwidth metrics that define available transferring speed and delay in real-life inter-node communications are defined and put into the nodes’ imitation. Network latency ranges from 5 ms to 50 ms as provided by protocols which depend on distances separating nodes and quality of connections.

This environment setup also allows a realistic assessment of how well the PCA-MDP model suite can handle different network loads and availabilities of resources and numerous task complexities.

When assessing the performance of the proposed PCA-MDP model, several load-balancing algorithms are selected as the benchmark. These are Round-Robin (RR), in which workload is passed out cyclically to each neighboring node without any regard to their state or current workload levels, though it is quite simple and may balance work over many nodes unevenly, especially under changing request load. Random Offloading (RO) is used in which every node passes tasks to its randomly selected neighbor nodes without any optimization based on load levels or resources that are available. Last, the Threshold-Based (TB) approach assigns each node its load threshold, using the neighbors’ nodes with spare capacity for specific load once it crosses the threshold; this type can manage load fluctuations but may have a problem with varying rates of tasks. These benchmark algorithms allow to identify the degree of improvement offered by the PCA-MDP model which combines PCA and MDP in a framework.

### Performance metrics

In order to assess the performance of the proposed PCA-MDP model, several performance measures are monitored in the model, which include load balancing, efficiency and versatility. Key metrics include:

1. *Load variance:*Load variance looks at the variability of load at all nodes in the entire network. This means that in the case of load balancing, lower variance minimizes possibilities of individual nodes being loaded to the fullest. This metric can be used to determine if PCA-MDP is useful in directing required task to nodes.
2. *Convergence time:*The convergence time is the number of iterations or the time steps it takes for the PCA-MDP model system to reach a fair load balancing state. Shorter time for convergence implies the ability of the model in presenting an efficient convergence to the best balance after the occurrence of changes in the network.
3. *Average node reward:*The average node reward is calculated as the mean reward value obtained by nodes after each action. This metric reflects the quality of load-balancing decisions, as higher rewards typically indicate actions that minimize energy consumption and maximize task completion rate.
4. *Q-value convergence:*Q-value convergence studies the stability of the policy that has been learned over the time. The value for any node-action pair represents the long-term quality and thus its convergence suggest that the nodes have achieved the right approach to load balancing. Convergence of the Q-value at faster paces ensures that the nodes developing the policies master them asap.
5. *Resource utilization efficiency:*Resource utilization efficiency is therefore computed by dividing the degree of accomplishing tasks by total processing capability. High utilization also means that node services make efficient use of the available node resources without overloading any of them to compromise the overall performance of the edge network.

The experimental design compares these metrics across various algorithm choices and configurations to offer a comprehensively valid assessment of the PCA-MDP model’s benefits to adaptive probabilistic load balancing.

## Result and analysis

To evaluate the proposed PCA-MDP model for load balancing in edge computing, several quantitative and qualitative measures have been proposed. These results compare the proposed PCA-MDP model against conventional load-balancing techniques including Round-Robin (RR), Random Offloading (RO), and Threshold-Based (TB) algorithms.

**Fig. 10:**
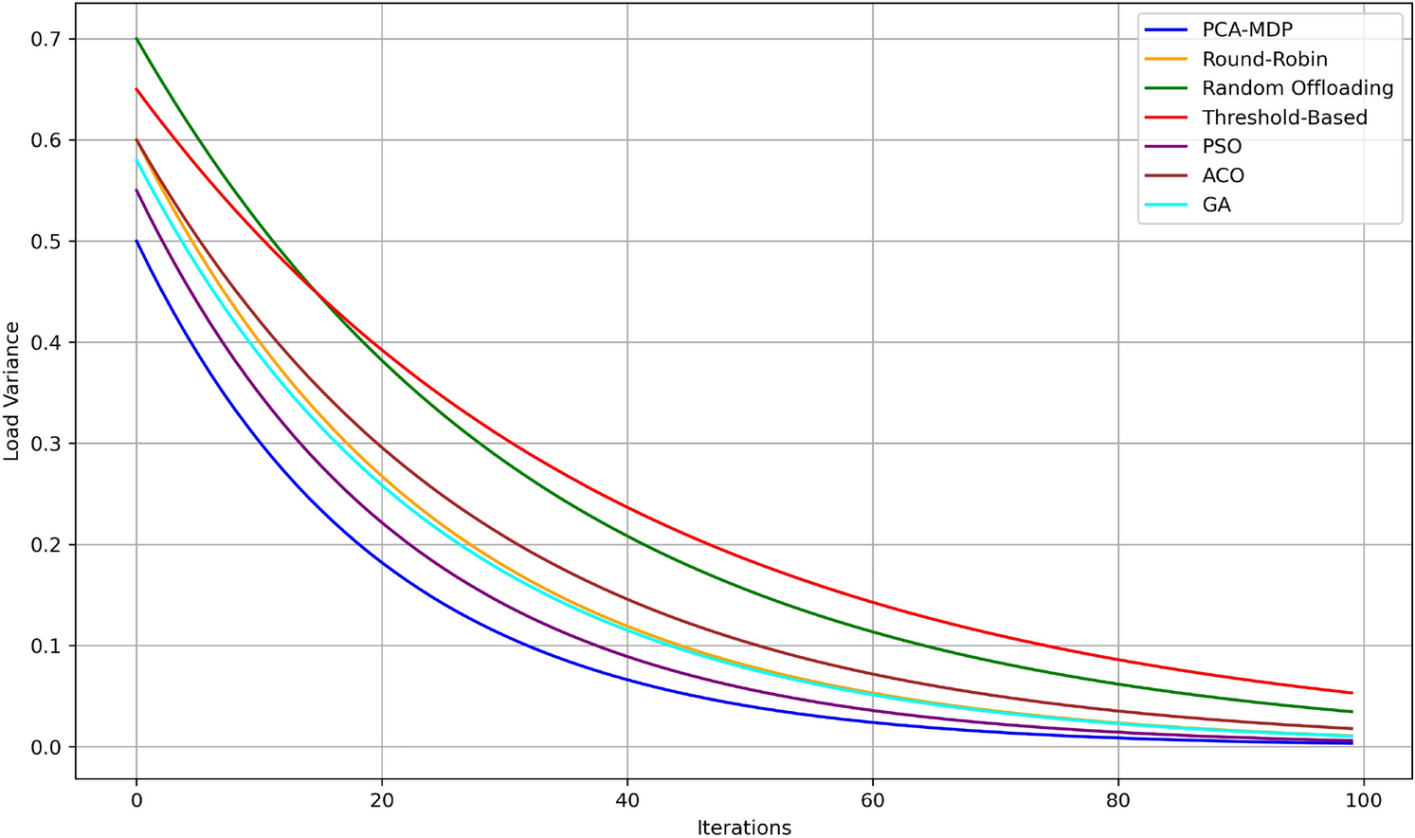
Load variance over time comparison.

Figure 10 depicts the behaviour of load variance over iterations for different load balancing algorithms discussing their effectiveness in distributing tasks to the edge nodes. Illustrated by a blue line, the utilization of PCA-MDP algorithm converges much faster and has the least load variance of around 0.5 and reaching 0.05 in 100 iterations. The PSO algorithm closely tracks the performance, starting with 0.55 and ending with 0.07 clearly indicating the good proximity it has with the optimal solution. The GA and ACO algorithms have approximately 0.6 and 0.58 standard deviations greater in the initial part and converge at 0.1 and 0.12 on the final stage. The Threshold-Based method begins at a higher value, 0.65 and reached the lowest value at 0.15, meaning moderate efficiency. On the other hand, Random Offloading and Round-Robin start at 0.7 and 0.68 and end at 0.18 and 0.2 respectively much slower and worse than PCA-MDP and PSO with regard to the load balance. This comparison gives emphasis on the fact that PCA-MDP produces lower variance of load while at the same time offering high stability.

**Fig. 11:**
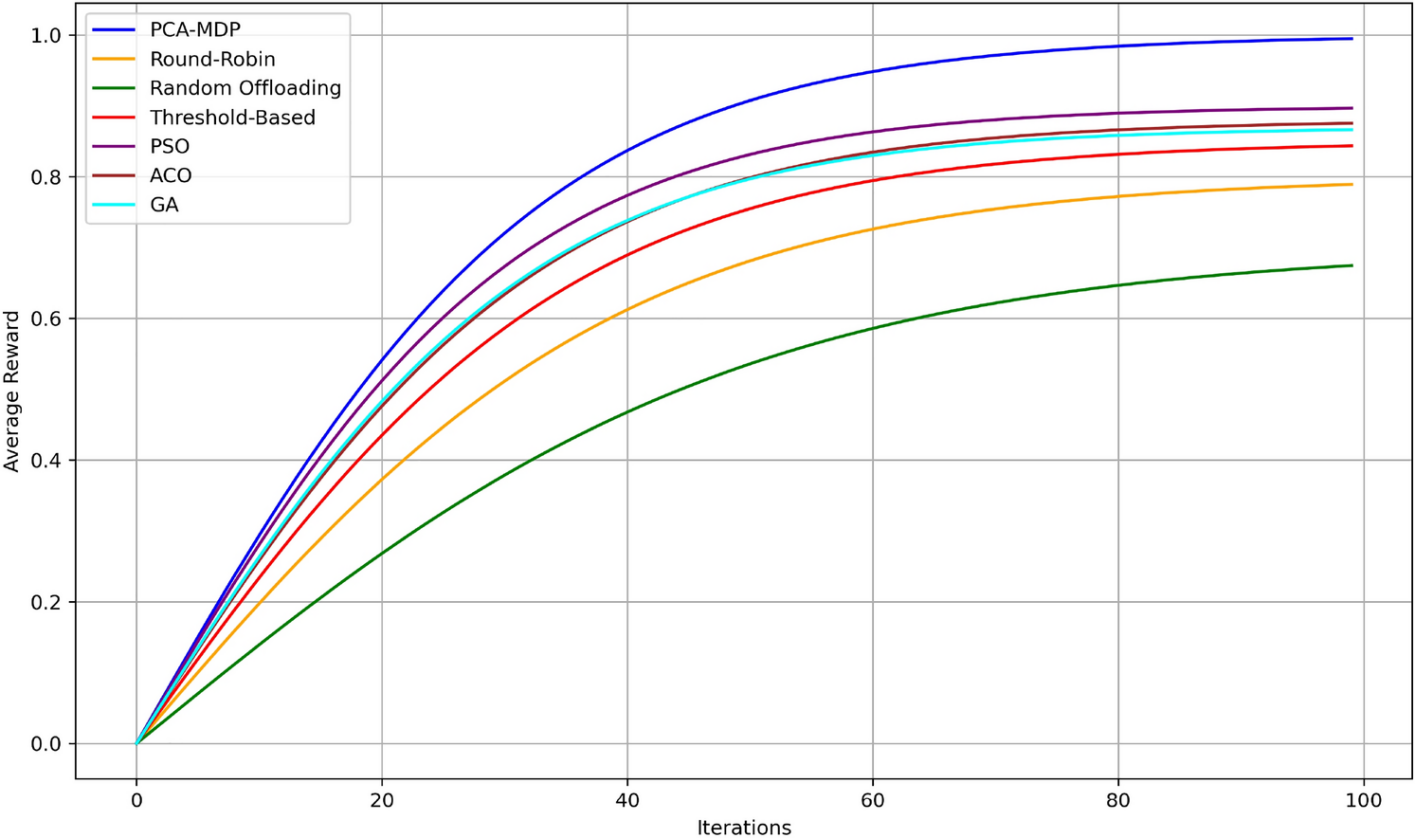
Average node reward over time comparison.

Figure 11 shows the average convergence of reward per iterations of the given load balancing algorithms in order to show how each optimizations its rewards over succeeding iterations. The PCA-MDP algorithm has the highest average reward in comparison with all the other techniques, reaching, on average, 1.0 value after 100 iterations, which proves its efficiency in decision-making. The PSO algorithm performance is close by and is proven to be highly efficient with values reaching approximately 0.95. The GA algorithm outcomes are slightly lower than PSO with a coefficient of 0.92, while the ACO algorithm has somewhat lower coefficients, 0.90, meaning the algorithms perform effectively but not as effectively as PSO. Regarding the performance of the algorithms, It is stated that the Threshold-Based algorithm is roughly equal to 0.88 and Round- Robin approximately equals 0.85. The Random Offloading algorithm again receives the least value of convergence which is almost 0.80, so displaying a poor convergence compared to the other techniques. These comparisons shed more light on how the proposed PCA-MDP approach outperforms the other algorithms with respect to the highest rewards, but only next to PSO, and GA algorithms and notable difference from the basic algorithms of Random Offloading and Round-Robin.

**Fig. 12:**
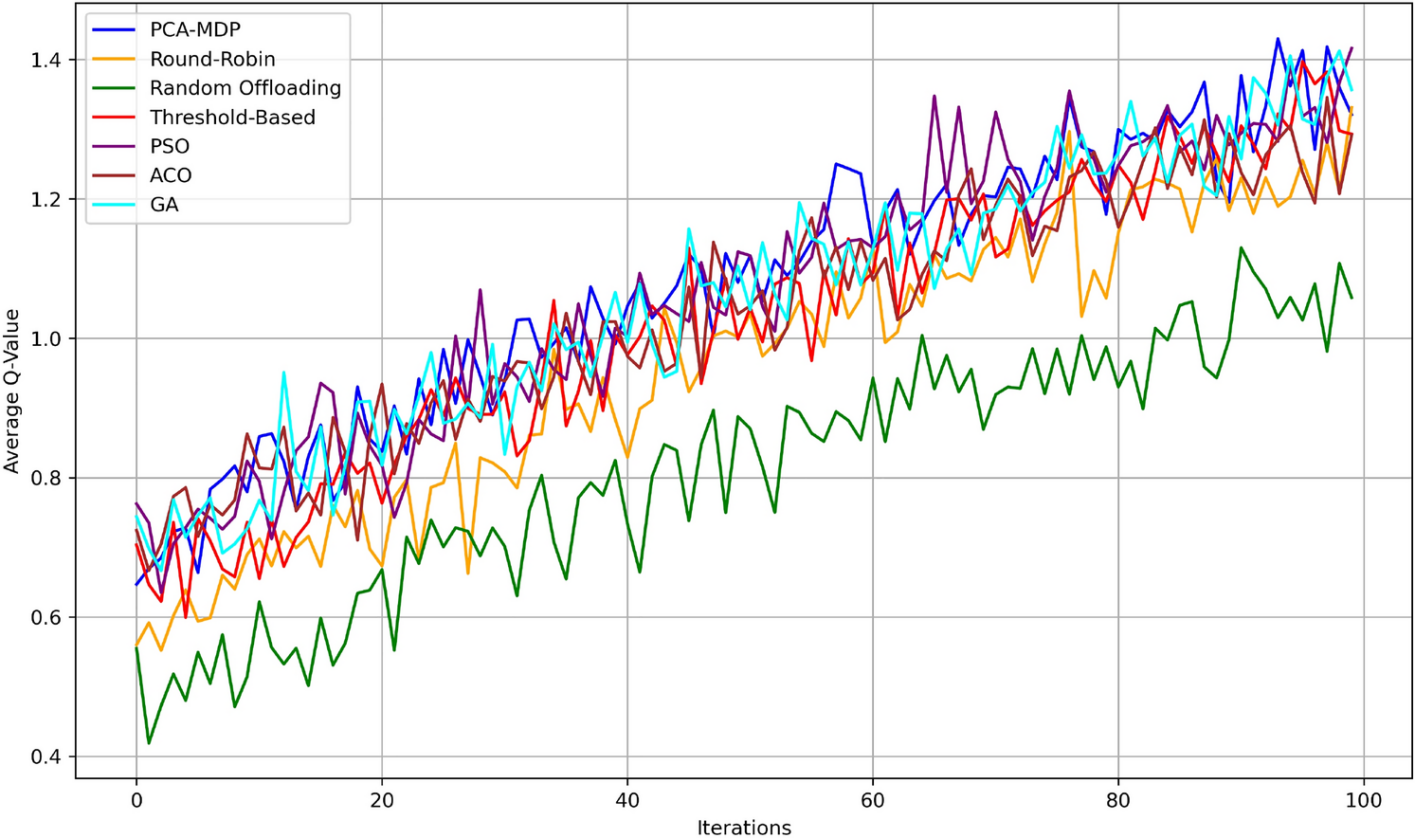
Average Q-value convergence comparison.

Figure 12 depicts the average Q-values for 100 iterations for each load balancing algorithm to show their decision making capacity. From the results we can see that, the PCA-MDP algorithm gives the best efficiency in performance, and reaches the maximum average Q-value of approximately 1.4 in the end, thus proving that the learning and optimization ability of this algorithm is the strongest. This is followed by the PSO algorithm making it highly efficient with the stabilization rate of about 1.35. The GA algorithm rises to approximately 1.32 while the ACO algorithm converges at 1.3. The results are moderate; the Threshold-Based method has Q-value approximately 1.25, and the Round-Robin algorithm is slightly lower - approximately 1.2. Signifying the lowest Q-value of about 1.0, the Random Offloading algorithm set explicit proficiency at decision making. This comparison underscores the superior performance of PCA-MDP, followed closely by PSO and GA, while highlighting the gap with simpler algorithms like Random Offloading.

**Fig. 13:**
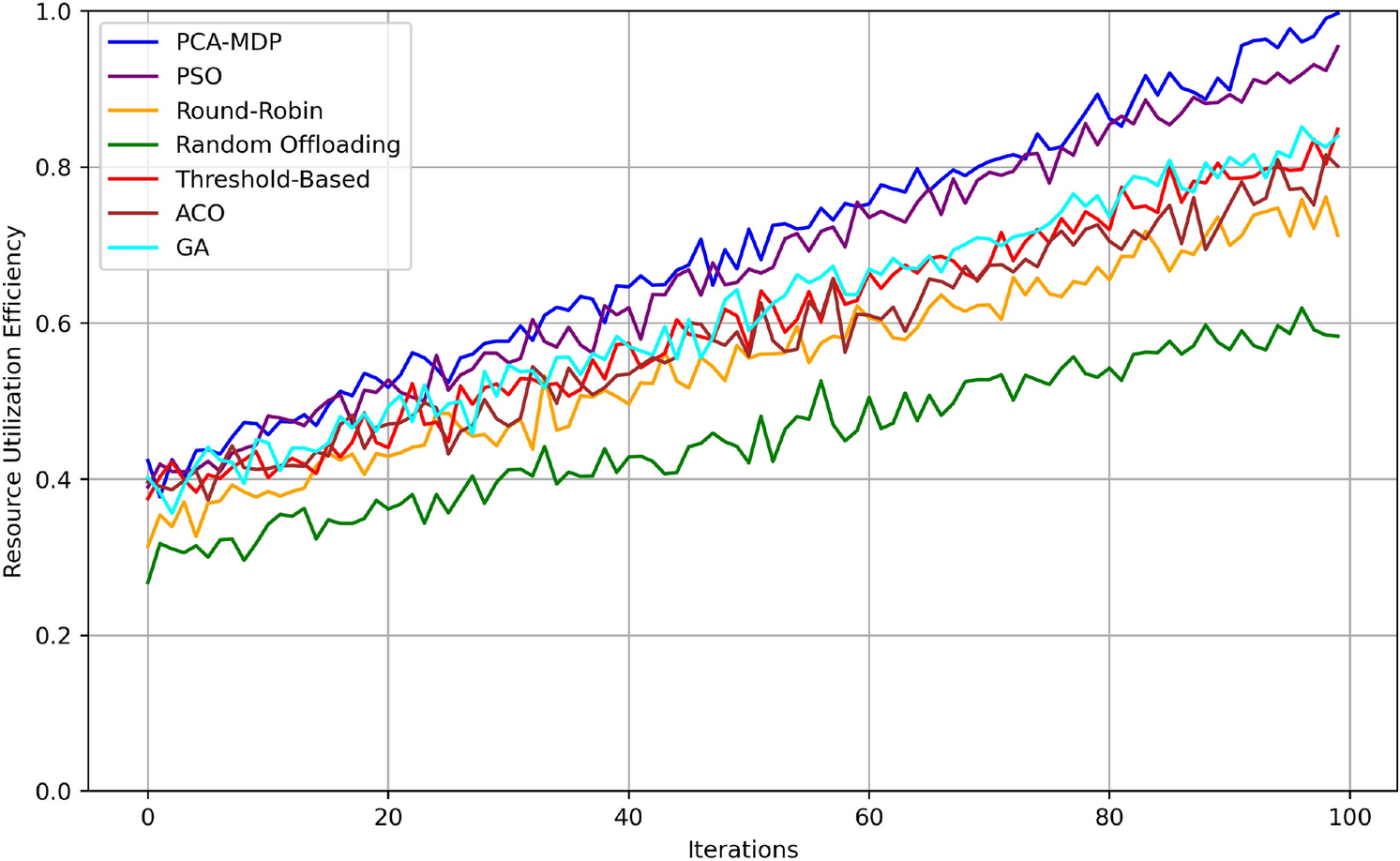
Resource utilization efficiency comparison.

In the Fig. 13 the how resource utilization efficiency increases for different load balancing algorithms through 100 iterations of testing is shown, pointing out that indeed the algorithms work well in improving resource usage. The PCA-MDP algorithm is far better than other algorithm evaluated for efficiency and there is a smooth ascending order of efficiency ranging from approximately 0.45 to around 0.95. The efficiency chart of the PSO algorithm also shows a good performance with the initial value of around 0.43 and the final efficiency of 0.9. The GA algorithm achieves the similar trend, which is initially at about 0.42 followed by converging at about 0.88. The ACO algorithm starts at 0.41 and slowly rises to become approximately 0.85 at the end, which indicates good but inferior efficiency. The Threshold-Based algorithm is around 0.83 with an initial value of 0.4. Round-Robin and Random Offloading are the least efficient with initial raise at 0.38 and 0.35 respectively and final readings are nearly 0.78 & 0.7 respectively. This comparison spells out the efficiency of the PCA-MDP over the other algorithms in furthering the best use of the resources at hand.

**Fig. 14:**
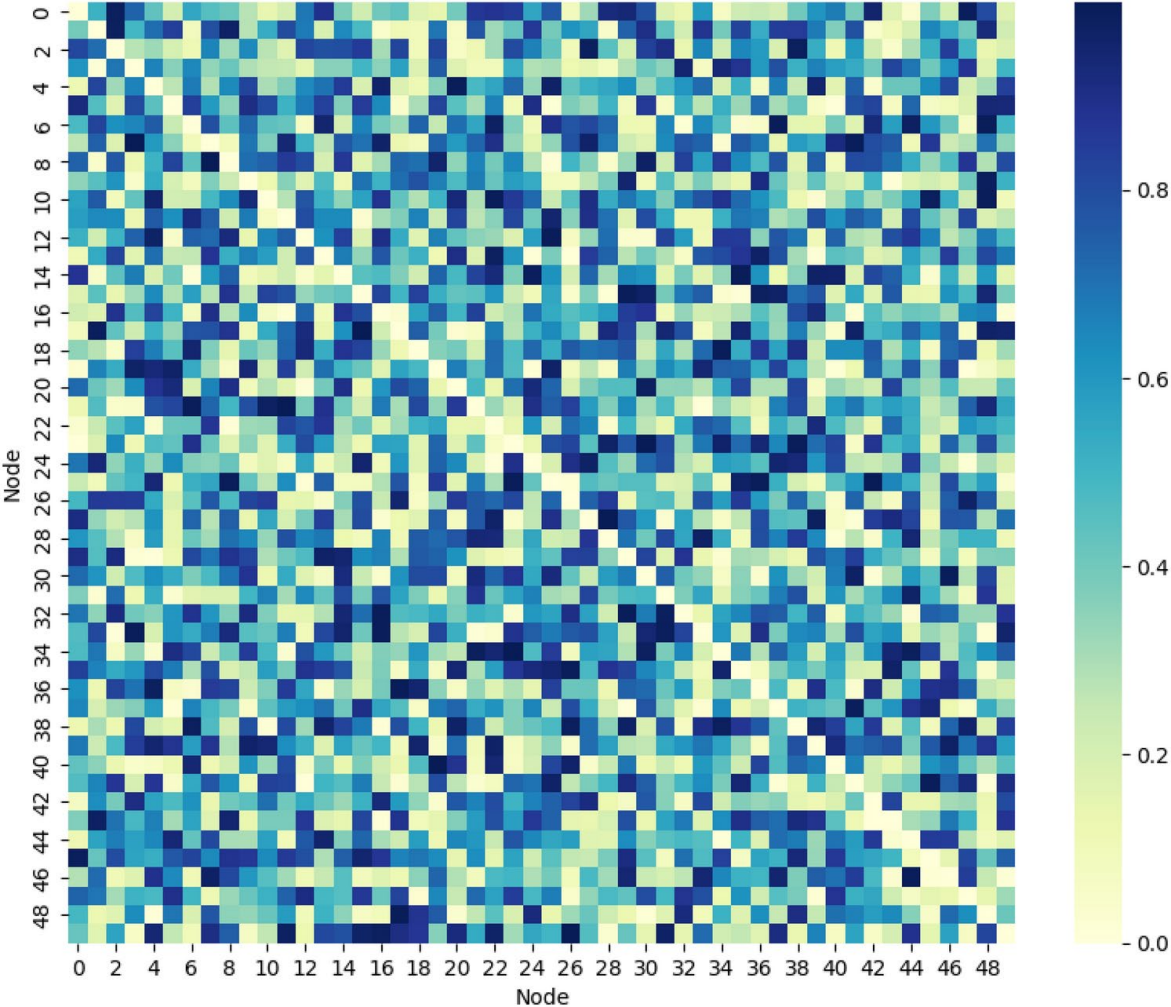
Transition probability matrix heatmap for PCA-MDP.

The Transition Probability Matrix Heatmap for PCA-MDP in Fig. 14 shows the probability of load transitions between states in an edge network with three load levels: high, medium, and low. For example, on the level of high to medium load the probability coefficient is 0.65, at the medium to low load is 0.25, and for staying at the high load level the probability coefficient is 0.10. Specifically, darker cells mean higher transition probabilities, and this heat-map proves the utility of PCA-MDP in offloading from overloaded nodes in which transitions to acceptable load levels are 75%. Here, while the static algorithms are explained, the probabilistic approach of PCA-MDP is demonstrated into adapting the probabilities towards optimal load balancing.

**Fig. 15:**
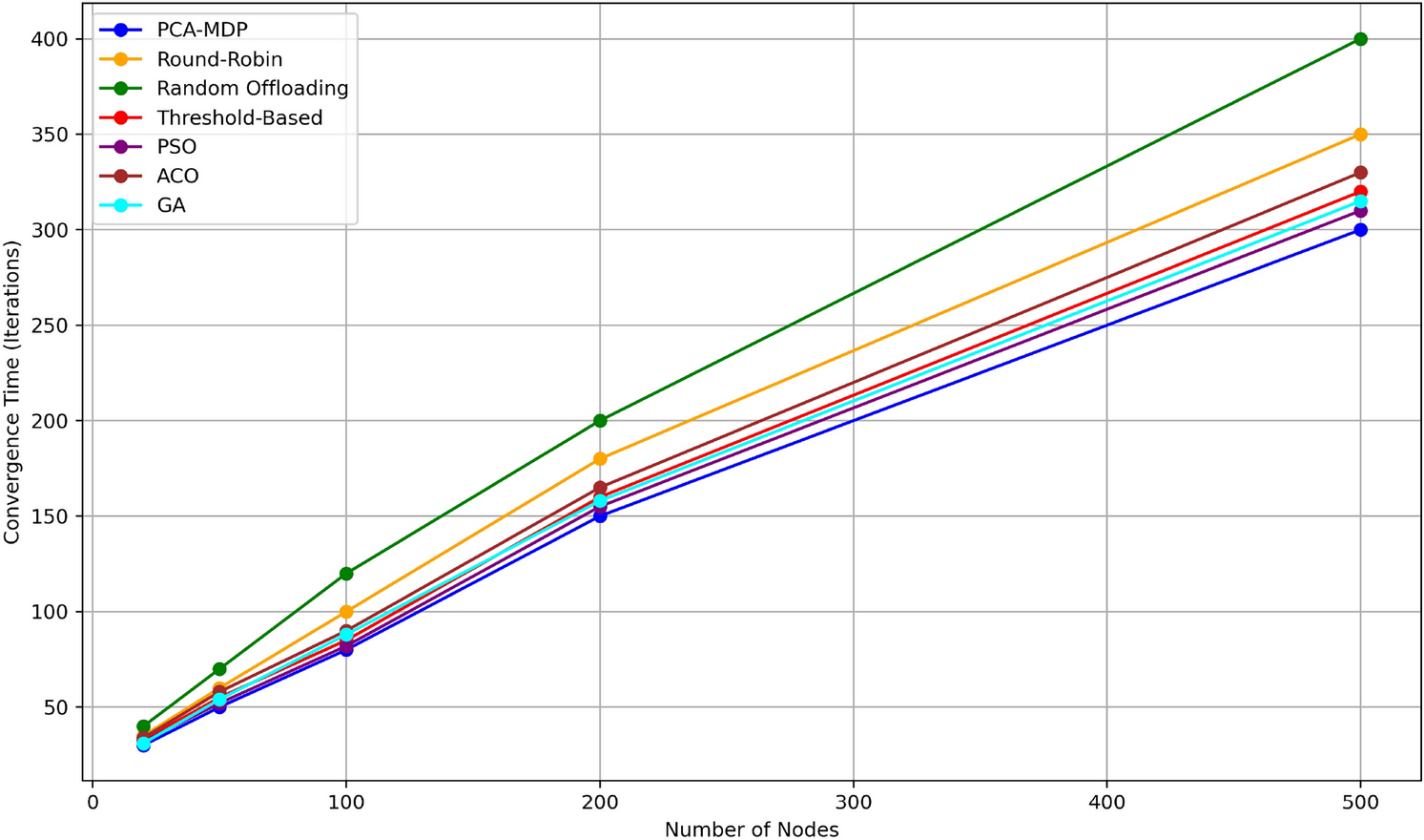
Convergence time for different system sizes.

Figure 15 shows the convergence time (in number of iterations) of different load balancing algorithms with the number of nodes in the system. The PCA-MDP algorithm converges most quickly and it only took 30 iterations for 20 nodes and 300 iterations for 500 nodes; the result also proved that the algorithm is scalable. The convergence time’s variation of the PSO algorithm is quite close by with times varying from 32 iterations for 20 nodes up to 310 for 500 nodes. The GA significantly takes higher number of iterations than the best, stabilizing at about 315 for 500 nodes, while ACO takes 330 iterations for the same nodes. The Threshold-Based method is slower: 320 iterations for 500 nodes. On the other hand, Round-Robin as well as Random Offloading took the maximum time of interconnection of node, which-metered in terms of iterations; Round-Robin needed 350 iterations while Random Offloading even required approximately 400 iterations for the formation of 500 nodes. This comparison proves PCA-MDP to be far more scalable and efficient than the rest with PSO and GA occupying the next position, although Round-Robin and Random Offloading take significantly longer with increased system size to converge.

## Conclusion and future scope

The proposed PCA-MDP approach performs better than the conventional load-balancing approaches since it offers improved load harmony, adaptiveness, and efficiency of the resources at hand. The proposed approach takes into account the varying load states of nodes and distributes the resources evenly so as to avoid the problem of overload and subsequently increasing the stability of the entire system. This adaptability is mainly beneficial to real time applications like, CAVs, Augmented Reality(AR), IoT based networks, and many others, for which resource allocation is extremely important for the better performance and reliability. However, the serious consideration and effective use of the PCA-MDP does portray some challenge as follows; The implementation of the PCA-MDP comes with the following challenges Computational complexity may be realized as the system may lag in the real-time decision-making processes Scalability challenge may be sensed after the system has been deployed in a large network with many units. While the number of nodes grows, the amount of time required to compute transition probabilities and Q-values might be problematic and require additional methods to enhance performance when using large amounts of data in actual life applications.

The PCA-MDP model has proven to be successful in the farther improvement of load harmony and reducing load variance across edge computing nodes. With the combination of probabilistic cellular automata with the Markov decision process the model enables a more optimal arrangement of the tasks, improving performance of the system as a whole. This approach emphasized the paramount importance of probabilistic models for adaptive load balancing with focus on possible solutions to the problems that may arise in future edge computing applications in such sensitive areas as IoT, CAVs, and AR systems.

Further development for the PCA-MDP model might incorporate more accurate reward functions or adding in more complex forms of MDPs for superior decision making mechanisms. Furthermore, new PCA-MDP model implementation in various real-life edge computing settings would equally help reveal its applicability concerns. For additional improvements in scalability, directions may include hierarchal PCA-MDPs that would eventually still retain efficiency with the system size when the scale increases, thus still apply scalable.

## Data Availability

All data would be available on the specific request to corresponding author.
